# Special Issue “Plant–Pathogen Interactions 2.0”

**DOI:** 10.3390/ijms262411889

**Published:** 2025-12-10

**Authors:** Jessie Fernandez, Sixue Chen

**Affiliations:** 1 Department of Microbiology and Cell Science, University of Florida, Gainesville, FL 32610, USA; 2 Department of Biology, University of Mississippi, Oxford, MS 38677, USA

Plant–pathogen interactions encompass a highly dynamic network of molecular events that define whether a plant successfully resists microbial attack or succumbs to disease. These interactions are driven by a continuous exchange of signals between hosts and microbes, beginning with the plant’s perception of potential invaders through surface receptors and intracellular sensors, and extending through to hormone-regulated signaling, transcriptional reprogramming, and metabolic adjustment [1,2,3]. Pathogens counter these defenses using diverse strategies, including the following: they deploy effectors that suppress immune responses, secrete enzymes that degrade structural barriers like the plant cell wall, rewire host molecular/cellular processes, such as protein trafficking and signaling, and exploit the host’s resources to support their growth and propagation [2,4]. At the same time, non-pathogenic and beneficial microbes colonizing the rhizosphere, phyllosphere, and endosphere are capable of activating overlapping defense pathways, modulating stress responses, and enhancing overall plant resilience [3,5,6]. The studies collated in this Special Issue illustrate many facets of this molecular ‘tug of war’, spanning the manipulation of salicylic acid (SA) metabolism and peptide-triggered immune regulation, the coordinated control of bacterial virulence systems, fungal reliance on protein-targeting and cell-wall-degrading enzymes, the viral hijacking of host cytoskeletal and nuclear components, and microbiome-mediated enhancement of plant defense and stress tolerance. Together, these nine contributions (eight research articles and one review) provide a timely and integrated view of plant–pathogen interactions, advancing our understanding of susceptibility genes, pathogen regulatory networks, effector biology, cell-wall degradation, viral movement, beneficial microbe-mediated defense activation, and rhizosphere-driven resilience. The contributions are expanded upon below.

Recent advances in identifying plant susceptibility factors illustrate that pathogens often exploit host metabolic pathways to suppress immunity. A compelling example is the characterization of peanut SA 5-hydroxylases *Ah*S5H1 and *Ah*S5H2, which catalyze the conversion of SA into 2,5-dihydroxybenzoic acid, thereby lowering SA levels and weakening SA-dependent defense signaling (Contribution 1). When expressed in *Arabidopsis thaliana*, both enzymes suppress SA-responsive *AtPR1* and *AtPR2* induction and increase susceptibility to *Pseudomonas syringae* pv. tomato DC3000. Differential tissue expression patterns in peanut further support their coordinated roles in modulating defense. These findings emphasize that susceptibility arises not only from pathogen aggression but also from host genes, the normal biochemical functions of which can be co-opted to weaken immunity.

The ability of plants to initiate multilayered immune responses is likewise exemplified by research on endogenous elicitor peptides. Immune activation mediated by the peach peptide *Pp*Pep2 involves a broad-pattern-triggered immunity-like response that is shaped in part by microRNAs, as revealed by miRNA-Seq analysis identifying 33 differentially expressed miRNAs. Most of the microRNAs change at 24 h after treatment, and they belong to families such as miR156, miR167, miR168, miR169, miR390, miR395, miR396, and miR482 (Contribution 2). Several of these miRNAs are associated with defense- and stress-related processes. For example, miR482 is linked to resistance (R) gene regulation, and miR395 is associated with sulfate-mediated protection against oxidative stress (Contribution 2). The authors further show that many predicted miRNA target transcripts exhibit opposite expression patterns following *Pp*Pep2 treatment, and that numerous targets are also regulated at the mRNA level in previously published *Pp*Pep2 transcriptome data [7]. Together, these findings indicate coordinated relationships between peptide-triggered gene expression and miRNA-mediated post-transcriptional regulation, demonstrating additional layers of complexity within plant immune signaling.

Insights into pathogen strategies are also expanded through work on bacterial regulatory networks. The identification of TfmR as a TetR-family transcriptional regulator in *Xanthomonas oryzae* pv. *oryzicola* illustrates how pathogens coordinate virulence through global control points (Contribution 3). This regulator positively regulates both the diffusible signal factor quorum-sensing system and the Type III secretion pathway by directly binding to a conserved promoter motif of *rpfG* and *hrpX*. Loss of TfmR impairs motility, extracellular polysaccharide production, hypersensitive response induction, and virulence, while constitutive expression of *rpfG* or *hrpX* partially restores pathogenicity (Contribution 3). These findings reveal that TfmR acts as an upstream integrator linking quorum-sensing cues with secretion system activation to optimize infection processes.

Multiple fungal studies provide complementary mechanistic perspectives on virulence determinants. In *Fusarium graminearum*, the role of the guided entry of tail-anchored (TA) proteins (GET) pathway in pathogenicity is demonstrated through the characterization of *Fg*GET3, an ortholog of the yeast (*Saccharomyces cerevisiae*) GET3 ATPase (Contribution 4). Functional conservation with yeast GET3 is demonstrated by the ability of *Fg*GET3 to restore normal function in a *S. cerevisiae* Δ*get3* mutant. Deletion of *FgGET3* results in pleiotropic developmental defects, including reduced hyphal growth, fragmented vacuoles, altered abiotic stress responses, markedly reduced conidiation, delayed conidial germination, and decreased deoxynivalenol mycotoxin production (Contribution 4). The Δ*FgGET3* mutant also exhibits significantly weakened virulence on wheat spikes. Together, these results underscore the significance of the GET pathway in targeting TA proteins and maintaining intracellular homeostasis, thereby supporting fungal development, secondary metabolism, and successful infection.

Polygalacturonase activity during barley infection by *Pyrenophora graminea* also emerges as a key virulence determinant. Four PG-encoding genes (*PgPG1-PgPG4*) were identified, with *PgPG1* showing the strongest inducible expression during infection (Contribution 5). Functional analysis via RNAi-silenced and overexpression strains revealed that silencing *PgPG1* reduced disease incidence by ~40–63%, whereas overexpression increased incidence by ~10–13%. *Pg*PG1 was confirmed to be secreted and, when transiently expressed in *Nicotiana benthamiana*, induced host cell death, supporting roles in pectin degradation and host response modulation (Contribution 5). Together, these findings reinforce the importance of cell-wall-degrading enzymes in fungal pathogenicity.

Effector biology continues to be a central theme in plant pathology, and this Special Issue includes a significant contribution in this area. A secreted CFEM-domain effector, *FvCFEM12*, is shown to be highly expressed during maize infection, to exhibit secretory activity, and to suppress Bax- and INF1-induced cell death in *N. benthamiana*, indicating strong immunosuppressive capabilities (Contribution 6). Heterologous expression of *FvCFEM12* in maize leaves using *P. syringae* strain D36E further compromises immune responses. Interaction with the maize wall-associated receptor kinase *Zm*WAK17ET is demonstrated, and silencing of this kinase increases susceptibility, highlighting its function as a host target of the effector (Contribution 6). Deletion of *FvCFEM12* impairs fungal virulence, alters colony and hyphal morphology, and reduces tolerance to cell-wall stressors, revealing a dual role that links fungal cell-wall integrity and physiology with immune evasion.

Plant–virus interactions are also represented. Using an in vivo co-immunoprecipitation strategy with an epitope-tagged alfalfa mosaic virus (AMV) movement protein (MP) expressed during genuine infection, Villar-Álvarez et al. (2024) developed a method to identify host factors associated with 30K family MPs in planta. This approach revealed 121 host protein candidates interacting with the AMV MP, including histone H2B, actin, 14-3-3A, the translation initiation factor eIF4A, and a peroxidase (POX) (Contribution 7). In vivo protein–protein interaction assays in *N. tabacum* show that H2B, actin, 14-3-3A, and eIF4A interact directly with multiple 30K family MPs, while POX is proposed to be part of a protein complex associated with these interactors (Contribution 7). Together, these findings point to a conserved reliance on core host components such as cytoskeletal elements, chromatin-associated proteins, and translation machinery to support intracellular trafficking, plasmodesmata-mediated movement, and viral spread.

This Special Issue further highlights how beneficial microbes can influence immunity and plant resilience. Genomic and transcriptomic analyses of *Burkholderia contaminans* strain BJ3 reveal a broad suite of plant growth-promoting traits, including pathways for indole 3-acetic acid (IAA) production, non-ribosomal peptides, volatile organic compounds, siderophores, hydrolytic enzymes, spermidine, nitrogen fixation, phosphate solubilization, and 1-aminocyclopropane-1-carboxylate deaminase (Contribution 8). In *A. thaliana*, BJ3 strengthens root architecture, increases lignin and antioxidant accumulation, accelerates flowering, and markedly improves tolerance to both salt and drought stress. Transcriptomic data shows activation of SA, ethylene, and IAA signaling networks, together with the induction of defense metabolites such as glucosinolates and camalexin, illustrating the capacity of beneficial microbes to simultaneously enhance stress adaptation and immune strength (Contribution 8).

A broader ecological perspective is provided through a conceptual review synthesizing rhizosphere-mediated strategies that enhance agroecosystem resilience (Contribution 9). Beneficial microbes, intercropping, green manures, and biochar amendments are described as tools that influence microbial community structure, soil carbon dynamics, nutrient cycling, and plant health. By integrating microbial ecology with agronomic management, the review highlights promising approaches for sustainable disease suppression, reduced reliance on chemical inputs, and enhanced plant resilience in the face of environmental change.

In summary, the articles collected in this Special Issue on “Plant-Pathogen Interactions 2.0” reflect the current landscape of research at the interface of molecular plant biology, pathology, and microbiome science. Together, these nine papers present a richly detailed and multidimensional view of plant–pathogen interactions. The studies demonstrate that pathogen infection relies on interconnected networks of regulatory proteins, effectors, metabolic pathways, and structural components, while plant defense emerges from hormonal balance, miRNA-mediated modulation, receptor signaling, and contributions from beneficial microbes. By combining molecular genetics, cell biology, systems-level approaches, and ecological perspectives, this Special Issue captures the diversity of contemporary research directions in the field and highlights promising avenues for developing durable resistance, microbiome-based protection strategies, and innovative solutions to crop health challenges.
